# Epigenetic Methylations on N6-Adenine and N6-Adenosine with the same Input but Different Output

**DOI:** 10.3390/ijms20122931

**Published:** 2019-06-15

**Authors:** Zhiqing Li, Ping Zhao, Qingyou Xia

**Affiliations:** 1 Biological Science Research Center, Southwest University, Chongqing 400715, China; zhaop@swu.edu.cn; 2 Chongqing Key Laboratory of Sericultural Science, Chongqing Engineering and Technology Research Center for Novel Silk Materials, Southwest University, Chongqing 400715, China

**Keywords:** epigenetic regulation, N6-methadenine, N6-methyladenosine

## Abstract

Epigenetic modifications on individual bases in DNA and RNA can encode inheritable genetic information beyond the canonical bases. Among the nucleic acid modifications, DNA N6-methadenine (6mA) and RNA N6-methyladenosine (m6A) have recently been well-studied due to the technological development of detection strategies and the functional identification of modification enzymes. The current findings demonstrate a wide spectrum of 6mA and m6A distributions from prokaryotes to eukaryotes and critical roles in multiple cellular processes. It is interesting that the processes of modification in which the methyl group is added to adenine and adenosine are the same, but the outcomes of these modifications in terms of their physiological impacts in organisms are quite different. In this review, we summarize the latest progress in the study of enzymes involved in the 6mA and m6A methylation machinery, including methyltransferases and demethylases, and their functions in various biological pathways. In particular, we focus on the mechanisms by which 6mA and m6A regulate the expression of target genes, and we highlight the future challenges in epigenetic regulation.

## 1. Introduction

Covalent modifications on nucleotides, including DNA and RNA, have expanded the genetic codes beyond their canonical bases to accomplish biological functions in multiple cellular processes in organisms [1].

At the DNA level, 5-methylcytosine (5mC) in CpG dinucleotides, the most abundantly modified DNA base, has been widely studied in eukaryotes and can regulate the expression of numerous genes by functioning as an epigenetic regulator [2,3,4,5]. Besides 5mC, more than 40 DNA modifications have been recorded in a newly established DNA modification database based on evidence or prediction. Unlike 5mC, the roles of those modifications in biological processes are few and still need to be defined further. One of them is the methylation on the sixth position of the purine ring in DNA adenine, also called N6-methadenine (6mA) [6]. Studies on 6mA methylation have drawn great interest from scientists during recent years. From 1968 and for a long time after, 6mA was only identified in bacteria [7] and was considered a unique modification in prokaryotes. Owing to technological breakthroughs, a large number of eukaryotic species has recently been reported to possess 6mA methylation, such as fungi [8], *Tetrahymena thermophile* [9,10], *Chlamydomonas reinhardtii* [11], *Caenorhabditis elegans* [12], *Drosophila melanogaster* [13], *Bombyx mori* [14], *Arabidopsis thaliana* [15], *Oryza sativa* [16,17], *Xenopus laevis* [18], *Danio rerio* [19], *Sus scrofa* [19], *Mus musculus* [18], and *Homo sapiens* [20]. Increasing evidence has thus uncovered genome-wide profiles of 6mA methylation and its function in regulating gene expression in eukaryotes [21].

At the RNA level, more than 100 distinct chemical modifications have been reported on RNAs, including messenger RNA (mRNA), transfer RNA (tRNA), ribosomal RNA (rRNA), microRNA (miRNA), small nuclear RNA (snRNA), and long non-coding RNA (lncRNA) [22,23,24]. Among these, N6-methyladenosine (m6A) is the most prevalent modification on RNA molecules. It was first discovered in 1974 in mRNA from cancer cells [25] and was subsequently identified in various species, such as viruses [26,27], bacteria [28], *Streptococcus pneumoniae* [28], *Saccharomyces cerevisiae* [29], *Thermus thermophiles*, *D*. *melanogaster* [30,31,32], *B*. *mori* [33], *Triticum aestivum* [34], *Zea mays* [35], *Avena sativa* [36], *A*. *thaliana* [37,38], *S*. *scrofa* [39], *M*. *musculus* [40,41], and *H*. *sapiens* [40,42]. Accumulating research results have supported the hypothesis of an important role of m6A modification in almost all processes of RNA metabolism [43].

In recent years, a large number of studies have pointed to the physiological functions of DNA 6mA methylation and RNA m6A methylation in organisms [44]. Here, we will focus on the current progress in research on these two kinds of methylation, with particular attention to the mechanisms by which DNA 6mA and RNA m6A regulate the expression of target genes.

## 2. Enzymes for DNA 6mA Methylation

**Figure 1:**
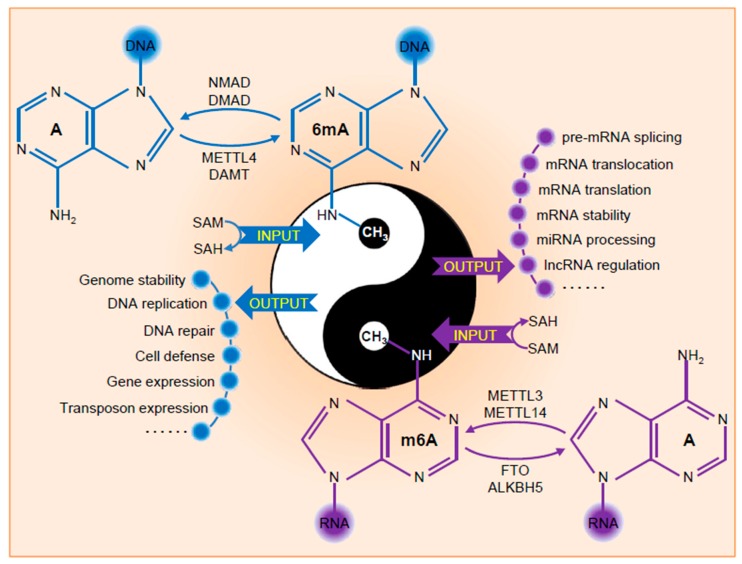
Modifications and functions of DNA 6mA and RNA m6A. Input represents the methylation process of adenine or adenosine by adding the methyl group to exocyclic NH2 at the sixth position of the purine ring through methyltransferase complex. Output exhibits the distinct functions of DNA 6mA and RNA m6A in multiple biological pathways.

DNA 6mA is generated by the addition of a methyl group from S-adenosyl-L-methionine (AdoMet/SAM) to exocyclic NH2 at the sixth position of the purine ring in adenine by specific methyltransferase enzymes (Figure 1) [21]. In bacteria, it is Dam and m.MunI that execute DNA adenine methyltransferase activities [45,46,47]. In eukaryotes however, only the DNA N6 adenine methyltransferase 1 (DAMT-1) in *C*. *elegans* has been reported to possess this activity in DNA adenine methylation. The evidence shows that the 6mA level was decreased by the depletion of DAMT-1 and increased by its overexpression (Figure 1) [12]. Further analysis showed that DAMT-1 contains an MTA70 domain, and this domain is widely present in eukaryotes [48]. Moreover, two homologs of this domain family, methyltransferase-like 3 (METTL3) and methyltransferase-like 14 (METTL14), have been confirmed as RNA m6A methyltransferases [49]. The possibility is thus raised that the other MTA70 domain proteins may have the potential for DNA 6mA methylation [50]. Indeed, it was shown that the DAMT-1 homolog in mammals is methyltransferase-like 4 (METTL4), one paralogue of METTL3 and METTL14 [48]. We also identified the homologous proteins of DAMT-1 and METTL4 in insects and the phylogeny analysis showed a high similarity of METTL4 among eukaryotic species [14]. RNA interference (RNAi)-mediated silencing of METTL4 expression in cultured *B*. *mori* cells resulted in a decrease of the 6mA level, which provided the first evidence of METTL4 functioning as a DNA 6mA methyltransferase (Figure 1) [14]. The contribution of METTL4 to mammalian 6mA methylation still needs to be clarified so as to confirm its conservation in enzymatic activity. However, a recent report in humans has shown that N-6 adenine-specific DNA methyltransferase 1 (N6AMT1) is able to catalyze DNA 6mA methylation, as evidenced by silencing and overexpression treatments [20].

Methylation on DNA adenine is a reversible process—the methylated 6mA can be removed by demethylases (Figure 1) [21]. Early reports showed that the alkylated DNA repair protein B (ALKB) family proteins could catalyze the demethylation of various methylated DNA and RNA nucleotides [51,52]. It has been demonstrated that N6-methyl adenine demethylase 1 (NMAD-1), one of the ALKB family members in *C*. *elegans*, is a 6mA demethylase, as indicated by the increase of the 6mA level by NMAD-1 depletion (Figure 1) [12]. By contrast, DNA 6mA demethylase (DMAD), the ten-eleven translocation (TET) homolog in *D*. *melanogaster*, which is generally considered a demethylase for 5mC methylation in mammals, has been revealed to have another role in removing 6mA methylation (Figure 1) [13,53]. In addition, the mouse and human ALKB homolog, ALKBH1, were shown to mediate DNA 6mA demethylation in mouse embryonic stem cells and human BEL-7402 cells, respectively [20,51]. Further, human fat mass and obesity-associated protein (FTO) was another 6mA demethylase in HEK293T cells, as evidenced by knockdown and overexpression experiments [54]. Except for the enzymes involved in the methylation and demethylation of DNA 6mA, the functional achievement of 6mA also needs other family proteins called “reader” proteins, that can recognize and identify 6mA marks, which is not discussed here.

Further biochemical and structural investigations on DNA 6mA methyltransferase and demethylase would provide more insight into our understanding of enzymes involved in DNA 6mA methylation in various species.

## 3. Enzymes for RNA m6A Methylation

Like in DNA 6mA methylation, the deposition of a methyl group also occurs from AdoMet/SAM to exocyclic NH2 at the sixth position of the purine ring in RNA adenosine (Figure 1) [55]. The catalyzed methyltransferases, however, are different from those in DNA 6mA methylation. Compared to the research on DNA 6mA methyltransferase, the methyltransferases involved in RNA m6A methylation have been more thoroughly investigated [48,49]. The enzymatic core for RNA m6A methylation is made of METTL3 and METTL14 proteins (Figure 1) [48,49]. As mentioned above, these two proteins contain conserved MTA70 methyltransferase domains, which are also essential for interactions between METTL3 and METTL14. The crystal structures of the METTL3–METTL14 heterodimer with MTA70 domains in the AdoMet-bound state further revealed that AdoMet can be present only in the METTL3 pocket and not in METTL14. From this it was concluded that METTL3 functions as the catalytic core subunit, while METTL14 plays a structural role for substrate RNA binding [56,57].

In addition to the METTL3 and METTL14 catalytic complex, many other proteins have also been reported to be involved in complex formation. For example, Wilms’ tumor 1-associating protein (WTAP) was identified as a METTL3/METTL14 binding partner but without catalytic activity; it is responsible for the recruitment of the METTL3/METTL14 complex to target RNAs [49]. Proteomic analysis of WTAP revealed that KIAA1429 was also responsible for the recruitment of the complex together with WTAP [58]. Recent work further showed that RNA binding motif protein 15 (RBM15) and its paralogue RBM15B were involved in the initial recruitment of the complex to its target site on a pre-mRNA [59]. Therefore, it was speculated that the proteins functioning in the recruitment of the METTL3/METTL14 complex to target RNAs would vary in different substrates and species.

The m6A can also be demethylated by m6A demethylases (Figure 1) [43,55,60]. However, only a few proteins have been identified to possess functional demethylase in several species. As of now, there are two candidate enzymes that have been considered to act as demethylases: FTO and ALKB homolog 5 (ALKBH5), both of which belong to the ALKB family proteins (Figure 1) [61]. FTO was first shown as a human obesity susceptibility gene and was found to influence gene expression through dynamic demethylation of m6A [62,63]. FTO downregulation induced an increase of m6A levels in HeLa and HEK293FT cells [64]. Furthermore, an FTO knockout mouse 3T3-L1 adipocyte lineage and mouse primary myoblast cells also increased the signals of m6A by dot blot analysis using an m6A-specific antibody [65,66]. Similar to FTO, depletion of ALKBH5 in HeLa cells increased m6A levels, whereas ALKBH5 overexpression decreased m6A levels [52]. In mice, a deficiency of ALKBH5 also increased m6A levels and resulted in abnormal spermatogenesis [52]. Although these proteins have been reported to be involved in m6A demethylation, they are only found in plants and metazoa (ALKBH5) or vertebrates (FTO) [55]. The absence of ALKBH5 or FTO in other species may mean that different enzymes evolved to demethylate m6A methylation. In addition to the above enzymes, several “reader” proteins for RNA m6A methylation have also been reported and are beyond the scope of this review.

## 4. Functions of DNA 6mA Methylation

6mA methylation was first identified in the genomic DNA of prokaryotes and was shown to function as an epigenetic mark that regulates several biological processes, such as DNA replication and repair, cell defense, and gene expression (Figure 1) [21,67,68]. The 6mA methylation sites and functions in eukaryotes, however, remained unexplored until very recently. Great progress in 6mA research came from the genome-wide mapping of methylation sites in three different eukaryotes, *C*. *reinhardtii*, *C*. *elegans*, and *D*. *melanogaster,* by immunoprecipitation-based high-throughput sequencing (MeDIP-seq) in 2015 [11,12,13]. This established the functional importance of 6mA methylation in eukaryotes.

In *C*. *reinhardtii*, DNA modifications on 5mC and 6mA have been reported for a long time [69,70], but the methylation sites and functions of 6mA had not been investigated. A recent report on genome-wide analysis of 6mA revealed that more than 14,000 genes (about 84% of all *C*. *reinhardtii* genes) were marked by 6mA methylation. Moreover, it was shown that this methylation is highly enriched around the transcription start sites of gene bodies and at gene promoters. Further gene expression analysis demonstrated the positive correlation between the presence of 6mA at promoters and the genes expressed actively [11].

Different from the presence of 5mC and 6mA in *C*. *reinhardtii*, the methylation on 5mC in *C*. *elegans* and *D*. *melanogaster* has long been a controversial issue [71,72]. It is widely accepted that there is a lack of 5mC methylation in these species. Studies on the identification of 6mA in *C*. *elegans* and *D*. *melanogaster* thus drew great interest from scientists [12,13].

In *C*. *elegans*, for the first time, DAMT-1 and NMAD-1 were identified to participate in the methylation and demethylation of 6mA respectively, providing evidence of the presence of 6mA in *C*. *elegans*. MeDIP-seq and single-molecule real-time (SMRT) sequencing revealed a broad distribution of 6mA across all chromosomes of the *C*. *elegans* genome [12]. Although the functional role of 6mA in *C*. *elegans* remains elusive, the crosstalk between methylations of adenine and histone H3K4 indicated a potential gene activation role of 6mA [12]. Indeed, further research on *C*. *elegans* after mitochondrial stress treatment revealed significant increases of global 6mA levels and demonstrated that histone H3K4me3 and 6mA are required for the transmission of mitochondrial stress adaptations to progeny [73].

In *D*. *melanogaster*, by using ultra-high-performance liquid chromatography-triple quadrupole mass spectrometry, coupled with multiple-reaction monitoring (UHPLC-MRM-MS/MS) analysis, 6mA methylation in *D*. *melanogaster* DNA was detected in very early embryogenesis and also in somatic tissues. Further in vitro and in vivo experiments identified that DMAD is able to regulate the demethylation of DNA 6mA modification. Genome-wide mapping of 6mA methylations between wild-type and DMAD mutants revealed that the main peaks of 6mA are enriched in the gene body of transposons, and 6mA methylation seems to suppress the expression of transposons (Figure 1) and nearby genes in *D*. *melanogaster* [13].

Thereafter, the presence of 6mA in many more eukaryotes has emerged in reports using MeDIP-seq and SMRT sequencing. In unicellular eukaryotic organisms, although *T*. *thermophile* was extensively investigated and found to contain 6mA [74], the lack of distribution information on genomic DNA interrupted its functional exploration. The recent mapping of 6mA in the *T*. *thermophile* genome thus exhibited that 6mA primarily occurs in the AT motif of the linker DNA regions in which the well-positioned nucleosomes and/or H2A.Z-containing nucleosomes are located. It was also shown that 6mA is widely present in the RNA polymerase II (Pol II)-transcribed genes, which may indicate its function in transcriptional activity [10].

In early-diverging fungal lineages, the identification and quantification of genome-wide 6mA from 16 phylogenetically diverse fungi were recently reported [8]. The contents of 6mA in these fungal genomes were significantly higher than those in other species: the methylated adenines were up to 2.8% of the totals, whereas other analyzed eukaryotes have only contained 0.05–0.2% of all adenines per genome. The genomic distributions of 6mA in diverse fungi also raised the possibility of regulatory roles in gene expression (Figure 1) [8,75].

In the lepidopteran *B*. *mori*, the potential 6mA methyltransferase METTL4 and 6mA demethylase NMAD were also identified. RNA interference (RNAi)-mediated silencing expression of METTL4 or NMAD affected the global levels of 6mA and disturbed the progression of the cell cycle. Further genome-wide analysis of 6mA methylation revealed its wide distribution in the *B*. *mori* genome, and the presence of 6mA appears to inhibit highly expressed genes [14].

In addition to invertebrates, 6mA methylations have been found in vertebrates as well. In *D*. *rerio* and *S*. *scrofa*, a large fraction of 6mA peaks are located in repetitive regions of the genome. The abundance of 6mA could cumulatively comprise 0.1–0.2% of all adenines during early embryogenesis, decreasing with the development of embryos [19]. In *X*. *laevis* and *M*. *musculus*, 6mA methylations have been shown to be extensively distributed across the genomes and are present in various somatic tissues. Genome-wide analysis of 6mA profiles indicated that 6mA peaks were more frequent within introns instead of exonic regions. Further gene ontology analysis revealed that genes with 6mA in *X*. *laevis* are involved in nucleic acid binding, metabolic processes, and transcription, whereas 6mA-targeted genes in *X*. *laevis* are associated with cell adhesion and ATP binding activity, which together suggest the distinct functions mediated by 6mA methylation in different species [18]. Additionally, upon environmental stress, the *M*. *musculus* brain showed elevated 6mA levels, which led to the upregulation of neuronal genes and downregulation of long interspersed element (LINE) transposon expression [76]. This exciting finding thus demonstrated a critical role of 6mA in the mammalian brain.

In *H*. *sapiens*, a very recent report confirmed the presence of 6mA in genomic DNA and identified the functional methyltransferase N6AMT1 and demethylase ALKBH1. Genome-wide mapping of 6mA showed that the density of 6mA is much higher in the mitochondria genome than those in autosomal chromosomes and sex chromosomes [20]. Moreover, significant enrichment of 6mA occurred in exon-coding regions rather than in introns, which is different from the reported profiles in *X*. *laevis* and *M*. *musculus* [18]. It was interesting that decreased genomic DNA 6mA methylation caused by silencing N6AMT1 or overexpressing ALKBH1 induced cancer cell growth and promoted tumorigenesis. These results raised great concerns about not only the distinct functions of 6mA in different eukaryotes but also the epigenetic regulation of 6mA for human cancers.

In addition, the investigation of 6mA has also obtained great breakthroughs in plants. In the model *A*. *thaliana*, global profiling of 6mA sites exhibited that 6mA methylations are widely distributed across the *A*. *thaliana* genome and enriched in the pericentromeric heterochromatin regions. Integrative analysis of 6mA methylomes and RNA sequencing data demonstrated that 6mA is associated with active gene expression [15]. Similar to *A*. *thaliana*, the contents of 6mA in *O*. *sativa* were 0.2% of all adenines and could be mapped to about 20% of genes and 14% of transposable elements [16,17]. All the data revealed the conserved distribution and function of 6mA methylation in plants.

Collectively, the recent studies on 6mA methylation in a number of eukaryotes have focused on the genome-wide mapping of 6mA distribution patterns. It was shown that the characterizations of 6mA in genomic DNA landscapes of different species are distinct, which may indicate the functional differentiation in various organisms. Further, the functions of 6mA in physiologic processes and the regulatory mechanism for gene expression in reported species are just beginning to be understood at present and require more research evidence in the future.

## 5. Functions of RNA m6A Methylation

RNA m6A methylation is the most prevalent modification on mRNA found in eukaryotes and is also detected in the other RNAs including rRNA, tRNA, miRNA, and lncRNA [22,23,24]. Compared to those of DNA 6mA methylation, the functions of RNA m6A methylation have been widely studied. In particular, the critical roles of enzymes-mediated regulation for target RNAs via the methylation and demethylation of m6A are well reported [48,55,77].

The first transcriptome-wide analyses of m6A sites were investigated in *H*. *sapiens* and *M*. *musculus* by using methylated RNA immunoprecipitation-sequencing (MeRIP-seq) in 2012 [40]. It was shown that there are 12,769 m6A peaks harboring 6990 coding gene transcripts and 250 non-coding genes in *H*. *sapiens* HepG2 cells, and there are 4513 m6A peaks within 3376 coding gene transcripts and 66 non-coding ones in *M*. *musculus* liver tissue. Enrichment analysis revealed that m6A methylations are frequently enriched in long internal exons and around the stop codon of genes. Interestingly, further silencing the expression of METTL3 in HepG2 cells elucidated an important involvement of m6A in regulating splicing events of multi-isoform genes and differentially spliced exons and introns. Moreover, the colocalization of METTL3 with splicing factors such as WTAP in nuclear speckles in *H*. *sapiens* HeLa cells provided the spatial possibility of m6A roles in splicing (Figure 1) [78,79]. Consistent with these findings, it was shown that the *H*. *sapiens* m6A is able to regulate alternative splicing of MyD88 in response to lipopolysaccharide-induced inflammation in dental pulp cells [80]. The regulation of alternative splicing by m6A was also reported in *D*. *melanogaster,* where about 2% of genes were regulated. It was confirmed that m6A is essential for Sxl gene splicing, and thus regulates sexual differentiation and prevents dosage compensation in females [30,31]. Increasing information on m6A mapping suggested interesting correlations that the enrichments of m6A on introns are related to slow splicing kinetics and the abundances on exons near splice junctions would be associated with fast splicing processing [81].

After the processing of gene splicing and the maturation of mRNA in the nucleus, the mRNA should be transported to the cytoplasm through the nuclear pore. During this phase, it was shown that m6A methylation on mRNA could be involved in its nuclear export (Figure 1) [48,82]. The evidence for this was that knockdown of WTAP disturbed the nuclear export of methylated mRNA [83] and silencing of METTL3 inhibited the pre-mRNA export of circadian clock transcripts and resulted in circadian period elongation [84]. Moreover, ALKBH5-deficient male mice showed increased m6A methylation in nuclear mRNA; the deficiency thus induced abnormal spermatogenesis and affected fertility [52]. Further research has revealed the mechanism that m6A methylation is essential for the recruitment of nuclear export factors such as NXF1 and NXT1 on mRNAs in order to promote nuclear export [85].

Recent findings have also uncovered the roles of m6A-mediated translation regulation on protein production (Figure 1), and this regulation showed either a positive or negative effect dependent on the target of the mRNAs. For instance, it was reported that METTL3 could recruit eIF3, a subunit of the translation initiation factor, to the 3′UTR in order to promote mRNA translation, and depletion of METTL3 decreased the translation of numerous m6A-containing mRNAs [86,87]. Besides the recruitment of eIF3, the interaction of METTL3 with CEBPZ, a transcription factor, in the promoters is able to promote translation as well [88]. However, it seems that the mechanism by which METTL3 regulates translation is different. The tethering of a catalytically inactive form of METTL3 in the 3′UTR does not affect its translational efficiency, whereas the catalytically active METTL3 is required in the promoter [86,88]. In addition to the positive regulation of m6A for translation, it could also negatively affect protein production. In *X*. *laevis* oocytes, the presence of m6A in the coding sequence (CDS) was associated with reduced protein levels, and this inhibition of translation was supported in mammalian cells [89,90]. Therefore, the regulation of m6A for translational efficiency would be distinct and complex in different species.

Apart from m6A regulation on mRNA for protein production, RNAi silencing of METTL3 in mammalian embryonic stem cells induced a longer half-life of numerous mRNA transcripts, which indicated the involvement of m6A methylation in mRNA stability (Figure 1) [91,92]. Studies further uncovered that m6A methylation may provide a marker for recruiting the cytoplasmic 5′−3′ exonuclease Xrn1 to promote rapid degradation of mRNAs [93].

In addition to the regulation of m6A on mRNA processes, m6A methylation has also been found to be involved in regulating the expression of miRNA and lncRNA. It was reported that the primary miRNAs with m6A methylation could be recognized by diGeorge syndrome critical region 8 (DGCR8), a subunit of the microRNA microprocessor complex, which is capable of recruiting the ribonuclease DROSHA to process pri-miRNAs (Figure 1) [94,95]. On the other hand, during human aging, differential m6A methylations were shown in peripheral blood mononuclear cells (PBMCs) from young and old cohorts. Interestingly, the transcript of argonaute-2 (AGO2), a catalytic protein in miRNA-mediated gene silencing, was highly methylated in young PBMCs and less so following aging [96]. Moreover, the expressions of miRNAs also fluctuated during the aging process. The evidence further showed the correlation of m6A methylated AGO2 mRNA with regulating its stability and miRNA expression, and it indicated a negative effect of m6A methylation on miRNA expressions during cellular senescence.

The research on m6A methylation on lncRNA has primarily focused on X-inactive specific transcript (XIST)-mediated dosage compensation. It was shown that the lncRNA XIST was heavily methylated with m6A, which resulted in the silencing of one of the two X chromosomes in female somatic cells (Figure 1) [59]. Indeed, knockdown of RBM15 led to the escape of X chromosome inactivation and the m6A methyltransferases, including METTL3, could bind to specific sites of XIST to promote the methylation of m6A [59].

As for m6A methylation on rRNA and tRNA, much work has been done in *E*. *coli* [97]. RlmF (YbiN), RlmJ, and KsgA are well-studied enzymes that are responsible for the methylation of adenosine in 16S and 23S rRNA [98,99,100]. Functional analyses of these rRNA methylations have demonstrated their roles in antibiotic resistance. Moreover, m6A methylation on rRNA may affect the structure of the rRNA itself, thus contributing to the assembly of ribonucleoprotein complexes. In addition to rRNA, m6A methylation on tRNA has only been reported in tRNA Valine in *E*. *coli* [97]. The methyltransferase for tRNA modification, however, has not yet been identified. Future work is also needed to discover the methylation on rRNA and tRNA in eukaryotes.

Altogether, m6A methylation has participated in almost all aspects of the RNA life cycle and is capable of influencing RNA metabolism. Emerging evidence on the biological relevance of m6A has also demonstrated important roles in cellular signaling pathways such as stem cell differentiation, DNA damage repair, and circadian rhythm. Importantly, current studies on m6A methylation have also established its association with the progression of a majority of human cancers. Further clarification of the regulatory mechanism of m6A in cancer development would provide novel diagnostic targets for clinical application.

## 6. Conclusions and Perspectives

During recent years, genome-wide mapping of DNA 6mA methylation and transcriptome-wide analysis of RNA m6A methylation have advanced our understanding of the roles of these two kinds of modifications in multiple biological processes in prokaryotes and eukaryotes. Although the regulatory mechanisms of 6mA and m6A on gene expression remain to be determined, emerging evidence has supported the hypothesis that the methylation profiles in which the methyl from SAM is added to DNA adenine or RNA adenosine are the same, whereas the consequences of 6mA and m6A methylations in terms of biological functions are very different.

The roles of 6mA and m6A in biological processes are largely ascribed to regulations on the distinct expression of target genes. A great number of genes have been reported as candidate targets of 6mA and m6A methylations via high-throughput sequencing in various species. For instance, there are about 23,000 genes harboring 6mA methylations and 7000 coding gene transcripts with m6A methylations in *H*. *sapiens*. The presence of 6mA on gene exons tends to activate the expression of genes, whereas transcripts of moderately expressed genes are more likely to be methylated by m6A, which suggests varied correlations between the expression levels of genes and the abundance of 6mA and/or m6A methylations. It was also shown that more than 5000 genes and 2000 genes in *B*. *mori* are methylated by 6mA and m6A, respectively. Interestingly, the current results showed a differential association of 6mA and m6A with gene expression in that the former is negative and the latter is positive in the case of the same targets. In the future, it would be worthwhile to investigate the crosstalk between 6mA and m6A methylations for gene expression in more species, as well as the correlation of target sites in different landscapes of genes. 

Functional studies on the enzymes involved in methylation of 6mA and m6A in several organisms have been limited. Although most species have been reported to possess the methylation of 6mA and/or m6A, their methyltransferases and demethylases remain to be explored. Therefore, the identification of functional enzymes in these species by recently developed techniques such as CRISPR/Cas9 would be critical to elucidating the methylation mechanism at the DNA and RNA levels. Moreover, the specific targeting of methylation sites mediated by methyltransferase or demethylase could also provide direct evidence for artificially regulating the expression of a given gene in organisms. As mentioned above, the crystal structures of RNA m6A methyltransferases have been best resolved. The structure of DNA 6mA methyltransferase such as METTL4, however, remains to be determined. The exploration of the structure of METTL4 will be an important step toward clarifying the biochemical characterization of binding and methylating DNA in detail.

In addition, as an important field in epigenetic regulation, DNA and RNA modifications are well-known aspects of the inheritance of epigenetic information. However, the transmission of epigenetic information via 6mA and/or m6A across generations is largely unknown. Future work is needed to decipher the mechanism of epigenetic regulation at both 6mA and m6A levels. Furthermore, upon developmental transitions or environmental stimuli, organisms must benefit from global changes of gene expression in a dynamic profile. Current studies have pointed to the potential regulation of 6mA and m6A signaling in these cues. It would also be interesting to uncover the mechanism of epigenetic inheritance on gene expression in response to diverse stimuli in forthcoming studies.

In conclusion, research of 6mA on DNA and m6A on RNA has recently received great attention and experienced rapid development. They are becoming novel hallmarks in the field of epigenetic inheritance. Further extensive investigations and important discoveries relating to their regulation and function in organisms will provide multiple insights and a more comprehensive understanding of the biology of 6mA and m6A methylations. Besides this, precision regulation of 6mA and m6A signaling in human tumorigenesis will be an exciting topic for cancer therapy.
